# The Pelagic Species Trait Database, an open data resource to support trait-based ocean research

**DOI:** 10.1038/s41597-023-02689-9

**Published:** 2024-01-12

**Authors:** Miram R. Gleiber, Natasha A. Hardy, Zachary Roote, Alana M. Krug-MacLeod, Caitlin J. Morganson, Zackary Tandy, Iris George, Cindy Matuch, Cole B. Brookson, Elizabeth A. Daly, Elan J. Portner, C. Anela Choy, Larry B. Crowder, Stephanie J. Green

**Affiliations:** 1 Department of Biological Sciences, University of Alberta, Edmonton, AB T6G 2R3 Canada; 2 California State University, Monterey Bay, CA 93955 USA; 3 Cooperative Institute for Marine Ecosystem and Resources Studies, Oregon State University, Newport, OR 97365 USA; 4 Scripps Institution of Oceanography, University of California San Diego, La Jolla, CA 92093 USA; 5 Hopkins Marine Station of Stanford University, Pacific Grove, CA 93950 USA

**Keywords:** Ecology, Zoology

## Abstract

Trait-based frameworks are increasingly used for predicting how ecological communities respond to ongoing global change. As species range shifts result in novel encounters between predators and prey, identifying prey ‘guilds’, based on a suite of shared traits, can distill complex species interactions, and aid in predicting food web dynamics. To support advances in trait-based research in open-ocean systems, we present the Pelagic Species Trait Database, an extensive resource documenting functional traits of 529 pelagic fish and invertebrate species in a single, open-source repository. We synthesized literature sources and online resources, conducted morphometric analysis of species images, as well as laboratory analyses of trawl-captured specimens to collate traits describing 1) habitat use and behavior, 2) morphology, 3) nutritional quality, and 4) population status information. Species in the dataset primarily inhabit the California Current system and broader NE Pacific Ocean, but also includes pelagic species known to be consumed by top ocean predators from other ocean basins. The aim of this dataset is to enhance the use of trait-based approaches in marine ecosystems and for predator populations worldwide.

## Background & Summary

Biological traits are increasingly used to characterize predator-prey interactions within changing ecosystems^1^. When combined, a suite of traits can be used to describe diet selection^2^ or identify prey guilds based on functional role^3^. Ultimately trait approaches seek to help scientists better predict interactions within ecological communities, especially in the scope of global change. In particular, habitat, behavior, morphology, and nutritional quality are important traits that can affect prey vulnerability across different aspects of the predation process (encounter, attack, capture)^4^. Habitat use (e.g., water column position) and migration behaviors impact encounter rates through spatiotemporal overlap, and schooling behavior can deter or facilitate predator attack. Morphological traits such as body shape and physical defenses influence the costs of prey capture, while body size affects consumption for gape-limited predators, and relative eye, fin or appendage size can influence predator detection and evasion^5^. Nutritional quality traits also mediate prey selection; predators select prey items in a manner that maximizes energy gain while minimizing energy expenditure^4^. Nutritional quality varies not only among species but also within species, reflecting geographic, seasonal, interannual, and longer-scale changes in environmental conditions^6^.

Understanding how species will interact with one another is important for predicting how ecological systems and services will be altered by forces such as climate change and biological invasions^7,8^. Trait-based approaches focus on the mechanistic drivers of ecological interactions and are emerging as a useful method for predicting variability in species distributions, community structures, and population dynamics under global change^9–11^. Further, identifying traits that recur across unrelated prey taxa offers a means to better anticipate predator resource use by simplifying complex foraging dynamics^3^. Assembling comprehensive databases of traits for biological communities facilitates ecological modeling of future species abundances, distributions, and food web structures^11,12^.

**Fig. 1:**
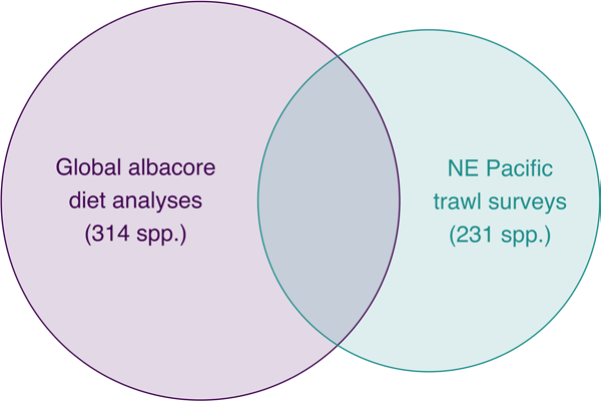
Venn diagram showing overlap in species among the datasets used to identify taxa for inclusion in the Pelagic Species Trait Database. NE Pacific trawl surveys = species observed in 15 years of annual National Oceanic and Atmospheric Administration (NOAA) midwater trawl summer surveys throughout the California Current System (CCS; 2005–2019), recent survey efforts by Fisheries and Oceans Canada (2017–2019) and the North Pacific Anadromous Fish Commission (2020). Global albacore diet analyses = species consumed globally by albacore tuna (*Thunnus alalunga*)^3^, including species consumed by albacore tuna in the CCS (2005–2019)^21–23^. Sample size for each species source is listed in parentheses.

This dataset^13^ contains traits for adults, juveniles, and larvae of 529 pelagic fish and invertebrate species found worldwide. Traits included describe 1) habitat use and behavior, 2) morphology and morphometrics, 3) nutritional quality (lipid, protein, energy density), and 4) population status information. The dataset was specifically created for its application in multi-facetted ecological modeling occurring in the California Current System (CCS) located within the NE Pacific Ocean. Therefore, species in the dataset are primarily from the CCS and broader NE Pacific Ocean to encompass both known and potential prey for pelagic predators^3^ (given anticipated future shifts in species distributions; Fig. 1). Globally important pelagic species known to be consumed by top ocean predators that are found in both the NE Pacific and other ocean basins (NW Pacific, Atlantic, Indian, Mediterranean) are also included to promote the use of trait-based approaches in marine ecosystems and predator populations worldwide. Detailed protocols are provided for trait data collection to serve as a framework for the expansion of this dataset in the future for other systems and predators.

**Fig. 2:**
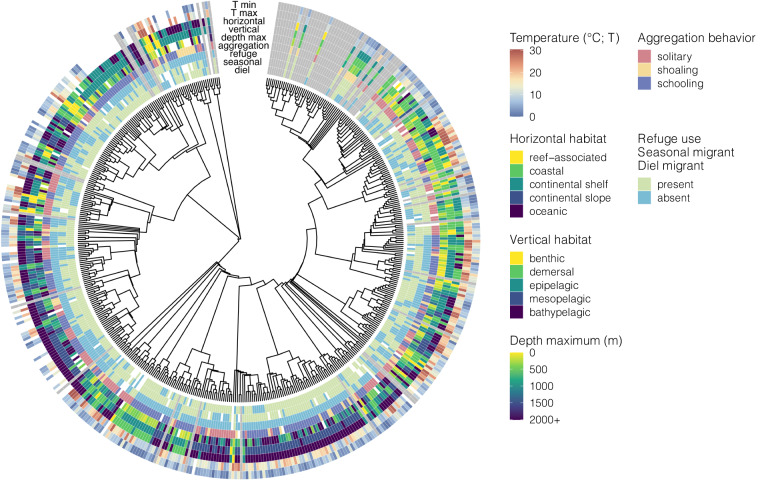
Phylogenetic distribution of habitat and behavioral trait data in the Pelagic Species Trait Database. Individual trait values are shown for adults of each species, although juvenile and limited larval information are also available in the dataset. White = species searched and no data found (NA), grey = species not searched in this dataset version (−9999). Traits are static for the species and lifestage.

**Fig. 3:**
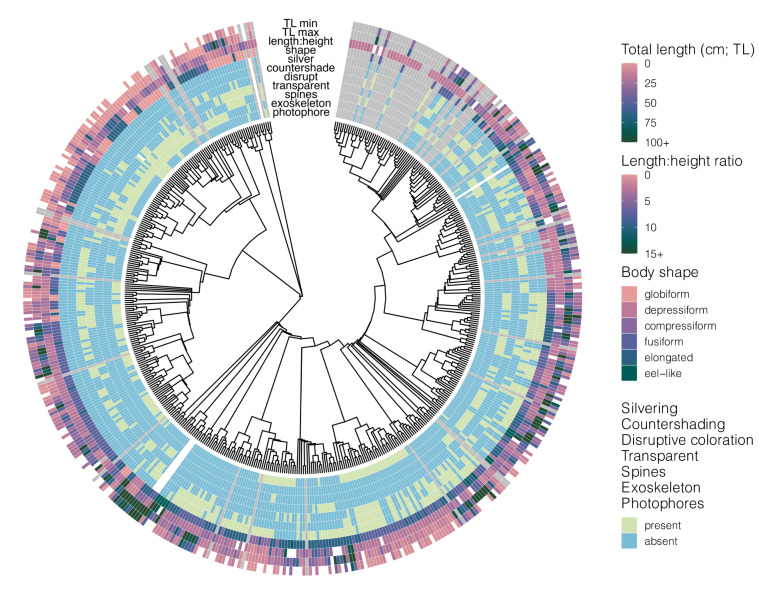
Phylogenetic distribution of morphological trait data in the Pelagic Species Trait Database. Individual trait values are shown for adults of each species, although juvenile information is also available in the dataset. White = species searched and no data found (NA), grey = species not searched in this dataset version (−9999). Length:Height ratios are mean values from multiple observations in the dataset, all other traits are static for the species and lifestage.

**Fig. 4:**
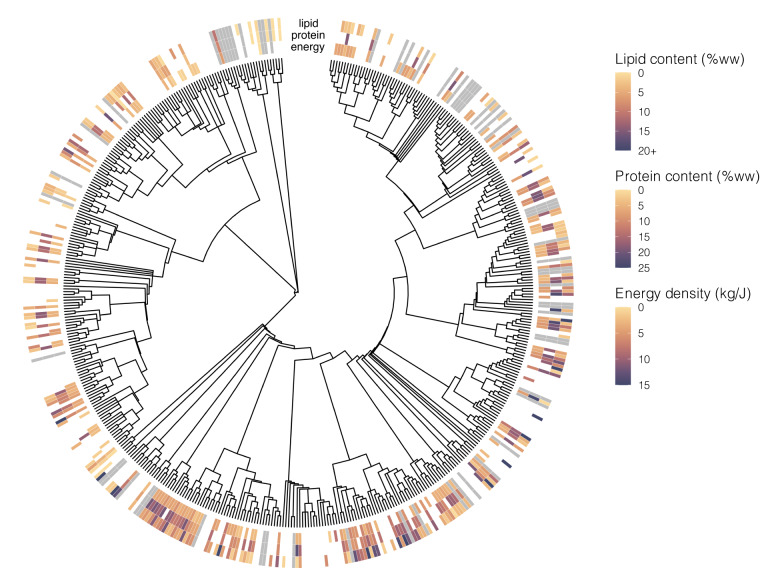
Phylogenetic distribution of nutritional quality trait data in the Pelagic Species Trait Database. Energy density, protein and lipid content are mean values from multiple observations in the dataset, integrating both adults and juvenile information. White = species searched and no data found (NA), grey = species not searched in this dataset version (−9999). %ww = percent wet weight.

**Fig. 5:**
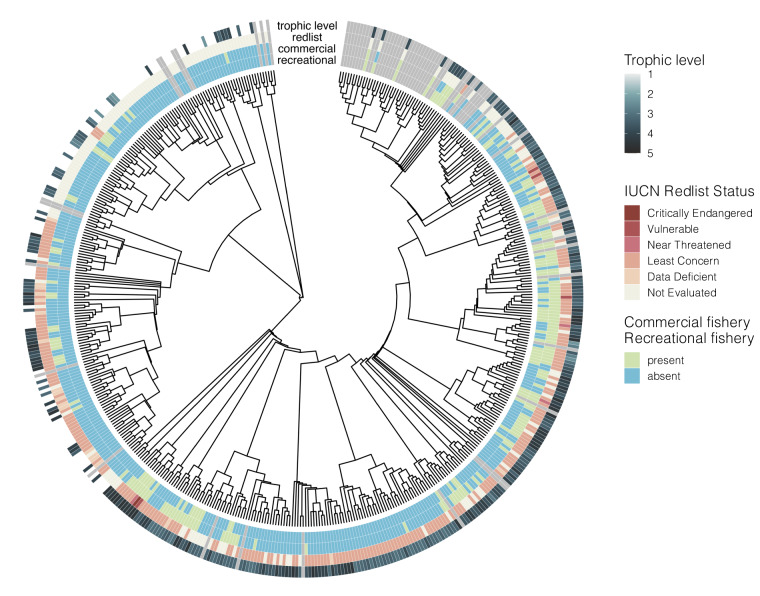
Phylogenetic distribution of population status trait data in the Pelagic Species Trait Database. Individual trait values are shown for adults of each species. White = species searched and no data found (NA), grey = species not searched in this dataset version (−9999).

With the publication of this trait dataset for pelagic species, we aim to encourage and facilitate the use of trait information in analysis of open-ocean ecosystem status and change, as well as enable pelagic systems to be a candidate for testing emerging trait-based analytical methods. In particular, the dataset as a whole serves as an opportunity to train and test statistical methods for trait imputation^14^. Knowledge gaps within the current dataset also emphasize directions for future work that further resolves trait classification analytically (Figs. 2–5). Of the species included in the dataset, 25% had complete records for all traits queried, while only 5% had less than half of the traits. Interestingly, species that are the focus of either commercial or recreational fisheries had information available for 95% of traits, while on average we were able to identify trait values for 87% of traits for non-fishery species. Nutritional traits are especially data poor, likely because values are generated from laboratory analyses that are time and resource intensive, requiring freshly collected specimens. Nutritional quality traits had the lowest data coverage (Fig. 4), with only 34% of species searched having protein content information, 41% for energy content, and 47% for lipid content. For this reason, this dataset augments literature searches with nutritional values for 55 CCS taxa from laboratory analyses (included in summary statistics), that fills prior data gaps in the region and globally.

## Methods

### Species list

The Pelagic Species Trait Database^13^ includes species representing pelagic communities of the CCS, as well as cosmopolitan species known to be important prey for pelagic predators in other ocean basins (n = 529; Fig. 1). For the NE Pacific, we included species observed in 15 years (2005–2019) of annual NOAA midwater trawls conducted in the CCS by the Southwest Fisheries Science Center Fisheries Ecology Division (SWFSC-FED) Rockfish Recruitment and Ecosystem Assessment Survey^15,16^, SWFSC Fisheries Resources Division (SWFSC-FRD) California Current Ecosystem Survey^17^, Northwest Fisheries Science Center Fish Ecology Division (NWFSC-FED) Stock Assessment Improvement Program (2005–2011)^18^, and NWFSC Coastwide Cooperative Pre-Recruit Survey (2011–2019)^19^. To encompass other communities in the NE Pacific we included species sampled by Fisheries and Oceans Canada (DFO; 2017–2019) and the North Pacific Anadromous Fish Commission’s International Year of the Salmon (2020). Beyond the NE Pacific, we included many known prey of a highly-migratory generalist predator, albacore tuna (*Thunnus alalunga*), compiled from a recent global meta-analysis of its diet^3^ (1880–2020). We note the dataset includes all species reported in the diets of *T. alalunga* collected in the CCS from 2005–2019^20–23^. Overall, species in this dataset represent 118 families of fish, 27 families of cephalopods, and 66 families of other invertebrates (e.g., crustaceans, jellies). Species names and phylogenetic information were verified using the Open Tree of Life^24^ and the World Register of Marine Species (www.marinespecies.org).

### Trait data collection

**Fig. 6:**
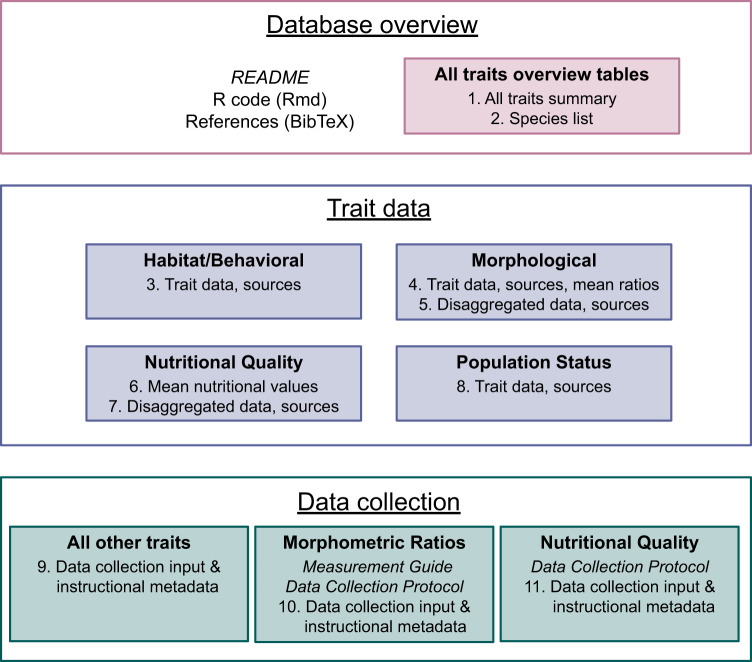
Overview of the design of the Pelagic Species Trait Database. Tables in .csv or .tab format are numbered according to the dataset file structure. Italics indicate .pdf file format, the R code is a .Rmd file, and references are included as a BibTeX file. Open rectangles indicate folders and shaded rectangles are subfolders in the file structure. Each Table (1–11) has an associated metadata table.

For each species we collected information on four trait categories: (1) habitat/behavior, (2) morphology (including morphometric ratios), (3) nutritional quality, and (4) population status. We searched online repositories (FishBase [www.fishbase.org] and SeaLifeBase [www.sealifebase.ca], Google Images [www.images.google.com]) and primary literature though bibliographic databases (Google Scholar [www.scholar.google.com], Web of Science [www.webofscience.com], Aquatic Sciences and Fisheries Abstracts [https://proquest.libguides.com/asfa], Federal Science Library Canada [https://science-libraries.canada.ca]) for species-level information and images. Search terms included scientific name or common name, lifestage (e.g. adult, juvenile, larva), and the trait of interest. When possible, trait data collection protocols followed standards outlined in the Preferred Reporting Items for Systematic Reviews and Meta-Analyses^25^, including consistent search terms, eligibility criteria for including data sources, data collection, source metadata, review, and bias reporting. We note that known trait information may change after data collection, especially for cryptic species and/or lifestages. All data manipulations, calculations, and summaries are described below and detailed in the R code included with the dataset^13^ (Fig. 6). Specific source information and notes on data collection are given in the sections below and reported for each trait per species and lifestage^13^ (Fig. 6, Table S1).

### Habitat/behavioral traits

Habitat/behavioral traits include vertical and horizontal habitat (categorical), depth and temperature range (numeric), aggregation, diel vertical migration, seasonal migration, and refuge use behaviors (binary; Fig. 2, Table 1). These traits were collected separately for adult, juvenile, and larval lifestages, and qualitative trait confidence level was noted based on the amount of available sources. If limited information was available for the species, adult traits were applied to the juveniles (but not larvae), unless specific information was found indicating a different value and it was reasonable that both lifestages likely occupy the same habitats^3^. Vertical and horizontal habitat use traits were directly recorded from online repositories and corroborated with species distribution maps and reported depth ranges from the primary literature when possible^3^. Where published literature expanded on, or differed from a general value reported by repositories, we used values from the published literature and data. For some traits, ordinal and binary versions were also included to facilitate future analyses.

**Table 1:** Overview of key traits included in the Pelagic Species Trait Database. Binary values are 1 = yes/present, 0 = no/absent; categorical values are listed in the description. Method lists the data collection search method used for each trait (L = primary literature, D = existing database, O = other online resource, I = image & measurements).

Trait category	Variable name	Variable description	Unit	Method
Habitat	depth_min	Minimum depth limit recorded for this species.	meters	L,D,O
	depth_max	Maximum depth limit recorded for this species.	meters	L,D,O
	temp_min	Minimum temperature limit recorded for this species	degrees celsius	L,D,O
	temp_max	Maximum temperature limit recorded for this species	degrees celsius	L,D,O
	vert_habitat	Primary vertical habitat association (benthic, demersal, epipelagic, mesopelagic, bathypelagic)	categorical	L,D,O
	horz_habitat	Primary horizontal habitat association (intertidal, reef-associated, coastal, continental shelf, continental slope, oceanic, freshwater)	categorical	L,D,O
Behavior	diel_migrant	Diel/diurnal vertical migration behavior.	binary	L,D,O
	refuge	Use of physical refuge	binary	L,D,O
	season_migrant	Seasonal migration behavior	binary	L,D,O
	gregarious	Primary aggregation trait (solitary, shoaling, schooling)	categorical	L,D,O
Morphology	body_shape	Body shape (eel-like, elongated, fusiform, globiform, compressiform, depressiform)	categorical	I
	l_min	Minimum length for the specific lifestage	centimeters	L,D,O
	l_max	Maximum length for the specific lifestage	centimeters	L,D,O
	defense_spines	Defensive spines present	binary	L,D,O,I
	exoskeleton	Presence of exoskeleton, carapace or armouring	binary	L,D,O,I
	transparent	Transparency present	binary	L,D,O,I
	col_disrupt	Disruptive colouration present	binary	L,D,O,I
	silver	Silvering present	binary	L,D,O,I
	countershade	Countershading present	binary	L,D,O,I
	photophore	Photophores present	binary	L,D,O,I
	TL_BH	Total length:body height morphometric ratio	ratio	I
	SL_BH	Standard length:body height morphometric ratio	ratio	I
	TL_TH	Total length:total height morphometric ratio	ratio	I
	SL_TH	Standard length:total height morphometric ratio	ratio	I
	SL_TL	Standard length:total length morphometric ratio	ratio	I
	eye_TL	Eye diameter:total length morphometric ratio	ratio	I
Nutritional quality	lipid	Lipid content in wet weight	percent	L
	protein	Protein content in wet weight	percent	L
	energy	Energy density in wet weight	kJ/g	L
Population status	trophic_level	Trophic Level (0–5); rfishbase covariate.	trophic level	D
	IUCN_status	IUCN Redlist status.	categorical	D
	commercial	Does the species have commercial value?	binary	D,O
	recreational	Does the species have recreational value?	binary	D,O

### Morphological traits and morphometric ratios

Morphological traits include lifestage-specific length range, body shape, and the presence and nature of defensive spines, exoskeleton, transparency, disruptive coloration patterns, silvering, countershading, and photophores (Fig. 3, Table 1). Morphometric ratios (relationships between body dimensions) are also included with the morphological traits to describe different aspects of body shape using continuous, numerical data, and were part of a separate data collection effort, which is detailed below. Morphometric ratios were only collected for adults and juveniles. Ratios were also used to convert different length types to total length, thus larvae lengths were unable to be converted to total length in some cases.

For each species and lifestage we quantified the relative total length (TL), standard length (SL), total height (TH), body height (BH), eye diameter, and dorsal fin height (Table 2). Measurements were taken from ~6 replicate images (range: 1–10) that were selected from the image search results based on a set of criteria to ensure accurate relative measurements. The criteria includes that images show the following: i) the correct species and lifestage, ii) the organism perpendicular to the frame of reference and not angled toward/away from the camera, iii) all dimensions measurable from the same image (lateral view for most organisms, dorsal view for flatfish, rays, crabs), and iv) soft-bodied organisms (e.g., cephalopods) with arms extended. When photographs or drawings from literature sources were not found we used the best available images. For some rare species and juvenile lifestages, the selection of images to choose from was limited and we were not able to adhere to all the criteria. Measurements based on any images that do not meet all the measurement criteria are noted. While morphometric ratios were lifestage-specific, we acknowledge that there is likely some variation within a lifestage that we are not capturing based on sample sizes and images available.

**Table 2:** Descriptions of length type measurements collected for different taxa in the dataset. The measurement guide included with the ‘data collection’ materials in the dataset further details these measurements.

Dataset measurement	Taxa	Measurement	Definition
Total length (TL)	fish	total length	Tip of the snout (or end of the longest jaw) to the end of the longest caudal lobe^38^
	cephalopod	total length	Tip of the tail (posterior end of mantle) to the end of longest arm; excludes the feeding tentacles which can retract^39^
	crabs	total length	Tip of longest pereopod on one side of body to tip of pereopod on other side of body
	other crustaceans	total length	Rostral margin (e.g., base of the antennae/setae) to the posterior margin of the telson excluding setae^40,41^
	gastropod	total length	Tip of the shell to the end of the body extended out of the shell
	other invertebrates	total length	Longest dimension (TL = SL)
Standard length (SL)	fish	standard length	Tip of the snout (or end of the longest jaw) along the lateral line to the base of the caudal fin (posterior limit of the hypural plate)^38,42^, where a groove forms
	cephalopod	mantle length	Tip of the tail (posterior end of mantle) to anterior most point of mantle^39^
	octopus	mantle length	Posterior tip of the mantle to the midpoint between eyes^39^
	crabs	carapace width	Distance between lateral spines of the carapace, at the widest point
	other crustaceans	standard length	Rostral margin to the base of the telson (posterior edge of 6th abdominal segment)^40,43^
	gastropod	shell length	Lateral distance from anterior to posterior on the shell (e.g., longest dimension)^44^
	other invertebrates	standard length	Longest dimension (TL = SL)
Total height (TH)	fish	total height	Tip of dorsal fin to tip of pelvic fin (or widest dimension to tips of fins), with fins fully extended (body height + fin lengths)
	cephalopod	total width	With fins (e.g., squid): diameter of mantle and fins combined, at the widest point; tip to tip of stabilizing fins. No fins (e.g., octopus): TH = BH
	crabs	total width	In dorsal view, anterior side of the folded claws to the posterior end of the carapace (carapace length plus folded claws)
	other crustaceans	total height	In lateral view, distance from dorsal-most margin to ventral-most margin at the widest point, including limbs
	gastropod	shell width	Shell width at widest point, including body out of shell (shell + appendages)
	other invertebrates	total height	Second longest dimension (TH = BH)
Body height (BH)	fish	body height	Base of the dorsal fin to base of pelvic fin; deepest part of the fish
	cephalopod	mantle width	Diameter of the mantle at the widest point (not including fins)
	crabs	carapace length	In dorsal view, distance from anterior to posterior ends of the carapace
	other crustaceans	carapace width	In lateral view, distance from dorsal side of the carapace to the base of the limbs
	gastropod	shell width	Shell width at widest point
	other invertebrates	body height	Second longest dimension (TH = BH)
Fin height (FH)	fish	fin height	Dorsal fin, from base to tip
	cephalopod	fin height	Stabilizing fin, from base to tip on one side
Eye diameter	all	eye diameter	Diameter of the eye, excluding tissue that contains the eye

Relative measurements for each image were collected in pixels, using ImageJ^26^ and measurements for each dimension were based on definitions from the literature (Table 2). SL is also used to describe the standardized length measurements for non-fish taxa (e.g. mantle length, shell length, carapace width). Morphometric ratios were calculated for each image as TL:SL, TL:TH, SL:TH, TL:BH, SL:BH, and eye diameter:TL. Fins can be folded or destroyed when individuals are removed from the water and/or preserved, thus fins were not always visible. In these instances, TH (includes dorsal and anal fins) or TL (includes caudal fin) could not be measured. Similarly, TL could not be measured if the arms (for cephalopod) or urosome (for crustaceans) were folded or not visible. Mean ratios were then calculated for each species and lifestage (n = 2–10), except in instances where only a single image was available. If species-specific morphometric ratios were not available, a proxy for the next available level of taxonomic identification (e.g genus, family) was used for the lifestage. Trait source information and notes on data collection are reported^13^ (Fig. 6, Table S1).

### Nutritional quality traits

For each species, we quantified lipid content (% wet weight, ww), protein content (% ww), and energy density (kJ/g ww) through a meta-analysis of published literature (Fig. 4). Keyword search terms include the scientific name, each nutritional quality metric (or synonym), and optional location keywords. To expand the search, we excluded quotations on some search terms to allow the search engine to also return results with synonyms (e.g., *percent* includes results for *proportion*). We also include data from laboratory analyses of energy density by bomb calorimetry and lipid and protein percentages by proximate composition for specimens collected in the CCS (see ‘Nutrional quality laboratory analyses’). Search results were evaluated for relevance using the title, abstract, keyword searches within the publication, and/or by visually scanning the paper. Lipid, protein, and energy density information were recorded to the highest level of detail reported in the publication, using individual values instead of mean values when possible. We recorded lipid and protein content in percent weight, and energy density in kJ/g, converting units as necessary. Research articles predominantly reported nutritional quality content as a proportion of ww, however dry weight (dw) and ash-free dry weight (afdw) data are also included in the literature. We standardized nutritional quality metrics as ww, converting dw and afdw as follows:

$$\begin{array}{c}{\rm{ \% }}\,{\rm{w}}{\rm{w}}=({\rm{ \% }}\,{\rm{d}}{\rm{w}}\,/\,100)\ast (100-{\rm{ \% }}\,{\rm{w}}{\rm{a}}{\rm{t}}{\rm{e}}{\rm{r}})\\ {\rm{ \% }}\,{\rm{w}}{\rm{w}}=((({\rm{ \% }}\,{\rm{a}}{\rm{f}}{\rm{d}}{\rm{w}}\,/\,100)\ast (100-{\rm{ \% }}\,{\rm{a}}{\rm{s}}{\rm{h}}\,{\rm{d}}{\rm{w}}))\,/\,100)\ast (100-{\rm{ \% }}\,{\rm{w}}{\rm{a}}{\rm{t}}{\rm{e}}{\rm{r}})\end{array}$$

Conversions use water (or moisture) content (%) and/or ash content (% dw) associated with the nutritional quality data reported in the paper. If these values were not reported with the dw or afdw values, the nutritional quality data was reported, but percentage ww could not be calculated. However, a water content proxy from the same species in the same region was used in a few invertebrates with consistently high water content (e.g., pyrosomes) or if a proxy has been used in the literature (e.g., krill).

In addition to nutritional quality information, we also reported covariates associated with each data point, when available. Covariates included sample size, moisture content, ash content, lifestage, age, sex, weight (ww mean, minimum, maximum), length (mean, minimum, maximum), temporal variables (sampling start/end year, month, day), and geographic location (latitude, longitude, ocean basin, descriptive location). Mean weight and length were estimated from the minimum and maximum values if not directly reported. Additionally, some location covariates were estimated if not reported (e.g., coordinates estimated from descriptive location), and details were noted. We standardized location information using Longhurst provinces, assigned by intersecting coordinates with Longhurst province polygons in R software^27^ (Version 3.6.0), or manually if only a general location was reported.

Mean nutritional quality metrics were calculated for each species using data (standardized to % ww) from 1) all global regions and 2) only values from the Pacific Ocean^13^ (Fig. 6, Table S1). We also include disaggregated, individual nutritional quality data in the dataset. Due to limited information about lifestage or age included in the literature, nutritional quality values from adults and juveniles are combined in mean values^13^ (Figs. 4, 6). Nutritional quality data was not collected for larvae, thus mean nutritional quality values were only applied to adults and juveniles.

### Nutritional quality laboratory analyses

#### Sample collection

Specimens were primarily collected during annual surveys performed by NOAA in the CCS. This includes the SWFSC-FRD California Current Ecosystem Survey from July - October 2021^17^, NWFSC Juvenile Salmon Ocean and Ecosystem Survey (JSOES) in May and June of 2016–2022^28,29^, NWFSC-FED Coastwide Cooperative Pre-Recruit Survey in May of 2016–2022^19^, NWFSC Newport Hydrographic Line (NHL) biweekly sampling from 2021–2022^30^, NWFSC Salmon Ocean Behavior and Distribution (SoBad) purse seine sampling effort in April of 2021^31^, NWFSC Fisheries Resource Analysis and Monitoring division (FRAM) sampling in June of 2022^32^, and NWFSC Cooperative research program quarterly survey collections from 2019–2022^33^. Additional specimens were collected during educational cruises associated with the Scripps Institution of Oceanography’s graduate courses (SIO295L Marine Biodiversity and Conservation, SIO277 Deep Sea Biology) in the summer of 2021 and 2022, and winter of 2022. Specimens were frozen and stored at −20 °C or colder until analysis.

#### Sample processing

Specimens were thawed, measured (standard, fork, or total length for fishes, mantle length for cephalopods, total length for crustaceans and other invertebrates; to the nearest mm), and weighted (nearest 0.01 or 0.00001 g for small samples). Sex and maturity were assigned based on visual inspection of the reproductive organs. To prepare samples for nutritional analysis, the whole individuals were either oven or freeze dried. In some instances, individuals were grouped across similar sizes, locations, and dates, and treated as a single sample to have sufficient dry material for analyses (n = 2–100’s for mesozooplankton and juvenile stages of crustaceans, 2–8 for juvenile fishes and cephalopods). For oven drying, samples were placed into a desiccating oven at approximately 60 °C for 2–3 days, until a consistent dry weight was achieved. For freeze drying, weighed samples were refrozen at −80 °C and placed in a benchtop freeze-dryer (FreeZone 2.5 L, Labconco, USA) for 3–7 days, until a consistent dry weight was achieved. Dry weights were recorded and moisture content was calculated for each sample.

Whole, dried samples were homogenized using either a mortar and pestle, coffee grinder, or tube mill (Tube mill 100 control, IKA, Germany; 25000 rpm for 30 second intervals), until a homogenous powder with consistent particle size was achieved. Dried tissues were stored in air-tight containers inside a desiccator for up to three weeks before being further processed or moved to long-term storage at −80 °C.

#### Energy density analysis using bomb calorimetry

Dried, homogenized samples were pressed into pellets (10–772 mg) using a Parr pellet press with a 3.75–10 mm die. The pressure applied to form each pellet was adjusted to prevent expressing oils out of the sample. The die and press were examined for expressed oil and cleaned with 95% ethanol between each sample. For some species with very high oil content (e.g., myctophids), pellets were hand-rolled to minimize oil loss. Specimens that when dried and pulverized formed a pellet of less than 0.02 g, were combined with benzoic powder and then pelletized for combustion with the energy of the added benzoic powder removed during the final calculation^34^.

Energy density was calculated by combusting pellets in a semi-micro calorimeter (6725, Parr Instruments, United States) with a water trap or an isoperibol calorimeter (C6000, IKA, Germany) without a water trap at either 22 °C or 25 °C. Both types of calorimeters used two decomposition vessels that were calibrated separately for each reaction temperature. Calibrations were checked at the beginning of each day by running 2–4 combustions of benzoic acid standard. Two replicates of each specimen were run then averaged together. When replicates differed by >8%, we ran a third replicate. To optimize consistency and accuracy of energy density measurements, benzoic acid standards tests were performed every 10–15 runs using a 200–1000 mg of benzoic acid.

#### Lipid and protein analysis using proximate composition

Total lipid and protein content was analyzed on a subset of specimens examined for energy density using the remaining dried, homogenized sample. Percent protein, lipid, dry matter and ash were determined in accordance with the standard methods of the Association of Official Analytical Chemists^35^. Carbohydrate percentage was not calculated, as it is negligible in these species. Total lipid content was determined by gravimetric analysis on 0.5 g of dry material using the Folch method with extraction using hexane (1 mL per sample). Nitrogen content was measured on a Leco C/N Analyzer using samples of 5–7 mg dry material and EDTA (Ethylenediaminetetraacetic acid) standards. A conversion factor of 6.25 was used to calculate crude protein from nitrogen content. Residual moisture content was measured following lipid extraction by heating lipid free tissue in an oven at 40 °C overnight. Ash content was determined by placing the dry tissue in a 120 °C furnace overnight. Replicate samples run for energy density (above) indicated adequate homogenization of all samples, thus replicates were unnecessary for lipid and protein analysis.

### Population status

Population status traits include trophic level, fisheries and conservation status (Fig. 5, Table 1). The trophic level is an estimate collected from FishBase and SealifeBase calculated by Ecopath software using the trophic levels of a predator’s known prey^36,37^. Trophic level is only available for adult lifestages of all fish species and some marine invertebrates. The fishery status was primarily collected from FishBase/SealifeBase, as well as online keyword searches for the species scientific or common name and the term “‘commercial”, “recreational” or “fishery”. The conservation status is collected from the IUCN Red List of Threatened Species (www.iucnredlist.org) for adults only.

## Data Records

The Pelagic Species Trait Database^13^ is publicly available on *Borealis*, an open-source repository in the Dataverse consortium maintained by a network of Canadian research universities, accessible by 10.5683/SP3/0YFJED. The dataset has three main components: 1) overview, 2) trait data, and 3) data collection files (Fig. 6, Table S1). The overview files include a README pdf, R code, BibTeX reference file, and summary tables with key trait variables (Table 1) for quick user access, and a species list with known geographic source information. Trait data modules include subfolders for each category of trait variables (habitat/behavior, morphology, nutritional quality, population status) with detailed references, expanded versions of variables (e.g., categorical, binary, ordinal), and data collection notes. Additionally, the trait categories ‘morphological’ and ‘nutritional quality’ each include a table with disaggregated data used to calculate mean morphometric ratios and nutritional quality values. The mean values calculated from these individual observations are reported in the summary trait table for each category (Table S1), and overview table (Table 1). Finally, the data collection information includes pdf files of protocols and raw data collection tables to support future collaborations to expand this dataset to other species and study systems.

Dataset files can be downloaded individually, or all together in a.zip folder with the structure described above (Fig. 6). Table S1 further details folders, filenames, and file descriptions. Tables are available for download in .csv or .tab format, and metadata tables are included with each detailing column descriptions, data types, and values. Tables have species, associated taxonomic classifications (e.g., Class, Order, Family, Genus) and lifestages as rows and their corresponding traits as columns. Missing information was labeled with ‘NA’, to identify gaps in the dataset. Some species were not included in data collection for all trait categories, these instances of traits not searched were labeled with ‘−9999’.

## Technical Validation

We used tiered steps during the data collection, processing, and sharing phases of dataset creation to validate data and ensure accuracy. First, all individuals performing data collection were trained through mentorship with a data collection supervisor. In total only 4–6 individuals performed data collection for traits, supervised by S.J.G, N.H., then M.R.G. over the creation of the dataset. This data collection team manually curated trait variables to ensure record accuracy performed through cross-checks between data collectors and often multiple data sources, with all references provided in the dataset. This collaborative process also included comparing interpretations of values found and assessing evidence in distilling ecological information to categorical and binary data types. If discrepancies were found, additional research was done or if information was indecisive an NA was assigned, with notes detailing the conflicting sources. This effort was version controlled through collaborative Google Sheets. The dataset files, including training materials such as protocols and tables for trait data collection, were compiled and reviewed by all data collectors and supervisors. These materials enable streamlined training of individuals to augment the dataset or fill in data gaps in the future.

Second, data processing involved outlier detection, cleaning of values, and comparisons of related traits. All numerical trait variables (e.g., nutritional values, morphometric ratios, depth, temp, etc.) were checked for outliers with frequency histograms. Values greater than two standard deviations from the mean were flagged and manually checked for validity against the original source to confirm they were correctly entered, and either retained, corrected or purged, as necessary. Morphometric ratio outlier values were re-measured on the original image, and values corrected, unless image issues were detected and data flagged as unusable. Categorical variables were cleaned during data processing to detect erroneous values and some variables checked by comparing relationships between related traits (e.g., body shape and morphometric ratios, vertical habitat and depth). All data processing steps were done in R software^27^ (Version 3.6.0), and version controlled through GitHub.

Finally, we provide an opportunity for dataset users to provide feedback through a guestbook feature. Our aim is to encourage engagement from users on maintaining the quality of this open-source dataset, thus we welcome any reporting of data errors, or suggestions on the dataset structure. We will incorporate these edits in updated versions of the dataset.

## Usage Notes

The Pelagic Species Trait Database is open source^13^ and publicly available on *Borealis*. The dataset is released under a CC-BY license permitting reuse with citation of this data descriptor, the dataset and any original sources, when possible. Users are requested to provide contact information prior to downloading to ensure updated versions are distributed to the user community, as well as enable solicitation of feedback from the community on the dataset design for user accessibility.

The dataset uses species names and phylogenetic information based on the World Register of Marine Species (www.marinespecies.org), thus users should confirm species names before querying the dataset for an accurate name match. Many traits were obtained from resources or literature indicating generalized characteristics of species and/or lifestages. We acknowledge that many traits are variable with environmental conditions, however this is not represented by the static trait variables. Users should reference the metadata files associated with data and data collection tables for descriptions of variables and collection information.

The R-script file provided with the dataset on *Borealis* details data manipulation, standardization, and calculations from initial data collections for the output files. This dataset is a static release, however as an evolving data product, successive versions may be released containing updates and corrections. Version 2 is used in this descriptor^13^; updated versions will be available on *Borealis*, accessed by 10.5683/SP3/0YFJED. Contact M.R.G. or S.J.G. for the status of dataset versions.

### Supplementary information

Table S1

## Data Availability

The R code used to process trait data is included with the Pelagic Species Trait Database files on *Borealis*^13^. Raw data inputs for the code are the data collection tables, with all other tables containing the processed, output data. Users are recommended to download the entire dataset, as the file structure is integrated into the code for data inputs.

## References

[1] McGill BJ, Enquist BJ, Weiher E, Westoby M (2006). Rebuilding community ecology from functional traits. Trends Ecol. Evol..

[2] Green SJ, Côté IM (2014). Trait-based diet selection: prey behaviour and morphology predict vulnerability to predation in reef fish communities. J. Anim. Ecol..

[3] Hardy, N. A. *et al*. Global synthesis of trait-based patterns in albacore tuna diets. *Fish and Fisheries*10.1111/faf.12807.

[4] Pyke GH, Pulliam HR, Charnov EL (1977). Optimal foraging: a selective review of theory and tests. Q. Rev. Biol..

[5] Green SJ (2019). Trait-mediated foraging drives patterns of selective predation by native and invasive coral-reef fishes. Ecosphere.

[6] Shalders TC, Champion C, Coleman MA, Benkendorff K (2022). The nutritional and sensory quality of seafood in a changing climate. Mar. Environ. Res..

[7] Poloczanska ES (2016). Responses of marine organisms to climate change across oceans. Front. Mar. Sci..

[8] Bartley TJ (2019). Food web rewiring in a changing world. Nat. Ecol. Evol..

[9] Laigle I (2018). Species traits as drivers of food web structure. Oikos.

[10] Brose U (2019). Predator traits determine food-web architecture across ecosystems. Nat. Ecol. Evol..

[11] Green SJ, Brookson CB, Hardy NA, Crowder LB (2022). Trait-based approaches to global change ecology: moving from description to prediction. Proc. R. Soc. B Biol. Sci..

[12] Gallagher RV (2020). Open Science principles for accelerating trait-based science across the Tree of Life. Nat. Ecol. Evol..

[13] Gleiber MR (2022). Borealis.

[14] Thorson JT (2023). Identifying direct and indirect associations among traits by merging phylogenetic comparative methods and structural equation models. Methods Ecol. Evol..

[15] Sakuma KM (2016). Anomalous epipelagic micronekton assemblage patterns in the neritic water of the California Current spring 2015 during a period of extreme ocean conditions. CalCOFI Rep..

[16] Santora JA (2021). Pelagic biodiversity, ecosystem function, and services: an integrated observing and modeling approach. Oceanography.

[17] Zwolinski JP, Stierhoff KL, Demer DA (2019). Distribution, biomass, and demography of coastal pelagic fishes in the California current ecosystem during summer 2017 based on acoustic-trawl sampling. NOAA Tech. Memo. NMFS.

[18] Phillips JA, Brodeur RD, Suntsov AV (2009). Micronekton community structure in the epipelagic zone of the northern California Current upwelling system. Prog. Oceanogr..

[19] Brodeur RD, Auth TD, Phillips AJ (2019). Major shifts in pelagic micronekton and macrozooplankton community structure in an upwelling ecosystem related to an unprecedented marine heatwave. Front. Mar. Sci..

[20] Glaser SM (2010). Interdecadal variability in predator-prey interactions of juvenile North Pacific albacore in the California Current System. Mar. Ecol. Prog. Ser..

[21] Glaser SM, Waechter KE, Bransome NC (2015). Through the stomach of a predator: regional patterns of forage in the diet of albacore tuna in the California Current System and metrics needed for ecosystem-based management. J. Mar. Syst..

[22] Madigan DJ (2015). Assessing niche width of endothermic fish from genes to ecosystem. Proc. Natl. Acad. Sci..

[23] Nickels, C. F., Portner, E. J., Snodgrass, O. E., Muhling, B. A. & Dewar, H. Juvenile Albacore Tuna (*Thunnus alalunga*) foraging ecology varies with environmental conditions in the California Current Large Marine Ecosystem. *Fish. Oceanogr.* **32**, 431–447, 10.1111/fog.12638 (2023).

[24] OpenTreeofLife (2019). Zenodo.

[25] O’Dea RE (2021). Preferred reporting items for systematic reviews and meta‐analyses in ecology and evolutionary biology: a PRISMA extension. Biol. Rev. Camb. Philos. Soc..

[26] Schneider CA, Rasband WS, Eliceiri KW (2012). NIH Image to ImageJ: 25 years of image analysis. Nat. Methods.

[27] R Core Team. R: A language and environment for statistical computing. (2022).

[28] Daly, E. A., Brodeur, R. D., Morgan, C. A., Burke, B. J. & Huff, D. D. Prey Selectivity and Diet Partitioning of Juvenile Salmon in Coastal Waters in Relation to Prey Biomass and Implications for Salmon Early Marine Survival. *North Pac. Anadromous Fish Comm. Tech. Rep*. 53–56 (2021).

[29] Daly EA, Brodeur RD, Auth TD (2017). Anomalous ocean conditions in 2015: impacts on spring Chinook salmon and their prey field. Mar. Ecol. Prog. Ser..

[30] Dumelle M (2021). Capturing copepod dynamics in the Northern California Current using sentinel stations. Prog. Oceanogr..

[31] Weitkamp, L. A., Bentley, P. J. & Litz, M. N. Seasonal and interannual variation in juvenile salmonids and associated fish assemblage in open waters of the lower Columbia River estuary. *Fish Bulletin* **110**, 426–450 (2012).

[32] Keller, A. A., Wallace, J. R. & Methot, R. D. The Northwest Fisheries Science Center’s West Coast Groundfish Bottom Trawl Survey: history, design, and description. *NOAA Technical Memorandum NMFS* (2017).

[33] Auth TD, Daly EA, Brodeur RD, Fisher JL (2018). Phenological and distributional shifts in ichthyoplankton associated with recent warming in the northeast Pacific Ocean. Glob. Change Biol..

[34] Churney KL, Armstrong GT (1968). Studies in Bomb Calorimetry. A New Determination of the Energy of Combustion of Benzoic Acid in Terms of Electrical Units. J. Res. Natl. Bur. Stand. Sect. Phys. Chem..

[35] AOAC. *Official Methods of Analysis of AOAC International*. (AOAC International, 2005).

[36] Pauly D, Christensen V (1995). Primary production required to sustain global fisheries. Nature.

[37] Pauly D, Christensen V, Dalsgaard J, Froese R, Torres F (1998). Fishing down marine food webs. Science.

[38] Kahn RG, Pearson DE, Dick EJ (2004). Comparison of standard length, fork length, and total length for measuring west coast marine fishes. Mar. Fish. Rev..

[39] Roper CF, Voss GL (1983). Guidelines for taxonomic descriptions of cephalopod species. Biol. Resour. Potential Cephalop. Mem. Natl. Mus. Vic..

[40] Morris DJ, Watkins JL, Ricketts CF, Buchholz F, Priddle J (1988). An assessment of the merits of length and weight measurements of Antarctic krill *Euphausia superba*. Br. Antarct. Surv. Bull..

[41] Guerao G, Díaz D, Abelló P (2006). Morphology of Puerulus and Early Juvenile Stages of the Spiny Lobster Palinurus Mauritanicus (Decapoda: Palinuridae). J. Crustac. Biol..

[42] Howe JC (2002). Standard length: not quite so standard. Fish. Res..

[43] Isaacs, J. D., Fleminger, A. & Miller, J. K. *Distributional atlas of zooplankton biomass in the California Current region: spring and fall 1955–1959*. (Marine Life Research Program, Scripps, 1969).

[44] Obaza A, Ruehl CB (2013). Regressions for estimating gastropod biomass with multiple shell metrics. Malacologia.

