# Supplementary material for: The Pelagic Species Trait Database, an open data resource to support trait-based ocean research
Source: Sci Data. 2024 Jan 12;11:2. doi: 10.1038/s41597-023-02689-9 (PMC10786825; doi:10.1038/s41597-023-02689-9)
Supplement: Supplementary file 1 — Table S1 [file 41597_2023_2689_MOESM1_ESM.docx]

**Supplementary Information**

**Table S1.** Directory structure inside the zip folder downloaded from The Pelagic Species Trait Database (https://doi.org/10.5683/SP3/0YFJED), hosted on Borealis. Includes folder names, file names, and descriptions. The general directory structure is also detailed in Figure 6.

| **Directory** | **Trait module** | **File name** | **Description** |
| --- | --- | --- | --- |
| overview | all_traits | 0_README Pelagic Species Trait Database Overview.pdf | README, documentation, and instructions file |
|  |  | references_Pelagic_Species_Trait_Database.bib | BibTeX reference file for all trait sources |
|  |  | Pelagic_Species_Trait_Database.Rmd | Instructional R code to provide a framework for the creation of future trait databases. |
|  |  | 1_pelagic_species_trait_database.csv | Key trait variables for each species and lifestage. Trait source information, notes, expanded variables, and individual replicates for mean values are detailed by trait type in Tables 3-8. |
|  |  | 1metadata_pelagic_species_trait_database.csv | Column descriptors and metadata for Table 1 (1_pelagic_species_trait_database.csv) |
|  |  | 2_trait_database_specieslist.csv | Source of species lists used to select species included in the database |
|  |  | 2metadata_trait_database_specieslist.csv | Column descriptors and metadata for Table 2 (2_trait_database_specieslist.csv) |
| trait_data | habitat_behavioral | 3_habitat_behavior_traits.csv | Habitat and behavioral traits for pelagic species. Includes source information for trait data as well as ordinal and binary versions of some variables. |
|  |  | 3metadata_habitat_behavior_traits.csv | Column descriptors and metadata for Table 3 (3_habitat_behavior_traits.csv) |
|  | morphological | 4_morphological_traits.csv | Morphological traits & morphometric ratios for pelagic species. Includes source information for morphological trait data, as well as ordinal and binary versions of some variables. |
|  |  | 4metadata_morphological_traits.csv | Column descriptors and metadata for Table 4 (4_morphological_traits.csv) |
|  |  | 5_morphometric_ratios_raw.csv | Individual relative body measurements and morphometric ratios for species with image data collection information and sources. |
|  |  | 5metadata_morphometric_ratios_raw.csv | Column descriptors and metadata for Table 5 (5_morphometric_ratios_raw.csv) |
|  | nutritonal_quality | 6_nutritional_traits.csv | Mean nutritional quality traits (lipid, protein, and energy density) for each species. Mean values are calculated separately for 1) all global regions, and 2) only values the Pacific Ocean. |
|  |  | 6metadata_nutritional_traits.csv | Column descriptors and metadata for Table 6 (6_nutritional_traits.csv) |
|  |  | 7_nutritional_raw.csv | Nutritional quality information for lipid, protein, and energy density content and associated metadata for pelagic species from published literature and unpublished data. |
|  |  | 7metadata_nutritional_raw.csv | Column descriptors and metadata for Table 7 (7_nutritional_raw.csv) |

| **Directory** | **Trait module** | **File name** | **Description** |
| --- | --- | --- | --- |
| trait_data | population_status | 8_population_status_traits.csv | Population status traits for pelagic species. Includes source information for trait data, as well as ordinal and binary versions of some variables. |
|  |  | 8metadata_population_status_traits.csv | Column descriptors and metadata for Table 8 (8_population_status_traits.csv) |
| data_collection | all_other_traits | data_collection_9_traits.csv | Initially collected trait data of habitat, behavioral, morphological, and population status variables. Provided as an example framework for future data collections. Metadata includes detailed instructions on data collection for each trait variable. |
|  |  | data_collection_9metadata_traits.csv | Column descriptors and metadata for Table 9 (data_collection_9_traits.csv) including detailed instructions for collection of each variable including categorical values, suggested sources, keyword search terms, additional materials, and functions/fields from the ‘rfishbase’ package for automated data extraction from FishBase/SeaLifeBase |
|  | morphometric_ratios | data_collection_10_morphometric_ratios.csv | Initial collected morphometric measurement data from species images. Provided as an example framework for future data collections. |
|  |  | data_collection_10metadata_morphometric_ratios.csv | Column descriptors and metadata for Table 10 (data_collection_10_morphometric_ratios.csv), including instructions for collection of each variable (and metainformation). |
|  |  | Morphometric Ratios Data Collection Protocol.pdf | Step-by-step instructions on building a data library of body shape ratios (body length : body height, standard length : total length, etc.) |
|  |  | Morphometric Measurement Guide.pdf | illustrated instructions on collecting body measurements for fish, cephalopods, crustaceans, and other marine invertebrates. Includes definitions of measurements used in the database. |
|  | nutritional_quality | data_collection_11_nutritional_quality.csv | Initially collected nutritional quality data from published literature and unpublished data. |
|  |  | data_collection_11metadata_nutritional_quality.csv | Column descriptors and metadata for Table 11 (ata_collection_11_nutritional_quality.csv), including instructions for collection of each variable (and metainformation). |
|  |  | Nutritional Quality Trait Data Collection Protocol.pdf | step-by-step instructions on building a data library of nutritional quality information (lipid, protein, energy density content). |
